# Graph neural network and diffusion model for modeling RNA interatomic interactions

**DOI:** 10.1093/bioinformatics/btaf515

**Published:** 2025-09-15

**Authors:** Marek Justyna, Craig Zirbel, Maciej Antczak, Marta Szachniuk

**Affiliations:** Institute of Computing Science, Poznan University of Technology, Poznan 60-965, Poland; Department of Mathematics and Statistics, Bowling Green State University, Bowling Green, OH 43403-0206, United States; Institute of Computing Science, Poznan University of Technology, Poznan 60-965, Poland; Department of Structural Bioinformatics, Institute of Bioorganic Chemistry PAS, Poznan 61-704, Poland; Institute of Computing Science, Poznan University of Technology, Poznan 60-965, Poland; Department of Structural Bioinformatics, Institute of Bioorganic Chemistry PAS, Poznan 61-704, Poland

## Abstract

**Motivation:**

Ribonucleic acid (RNA) function is inherently linked to its 3D structure, traditionally determined by X-ray crystallography, Nuclear Magnetic Resonance, and Cryo-EM. However, these techniques often lack atomic-level resolution, highlighting the need for accurate *in silico* RNA structure prediction tools. Current state-of-the-art methods, such as AlphaFold3, Boltz1, RhoFold, or trRosettaRNA, rely on deep learning models that represent residues as frames and use transformers to learn relative positions. While effective for known RNA families, their performance drops for synthetic or novel families.

**Results:**

In this work, we explore the potential of graph neural networks and denoising diffusion probabilistic models for learning interatomic interactions. We model RNA as a graph in a coarse-grained, five-atom representation and evaluate our approach on a dataset of small RNA substructures, known as local RNA descriptors, which recur even in non-homologous structures. Generalization is assessed using a dataset partitioned by RNA family: the training set consists of rRNA and tRNA structures, while the test set includes descriptors from all other families. Our results demonstrate that the proposed method reliably predicts the structures of unseen descriptors and effectively adheres to user-defined constraints, such as Watson–Crick–Franklin interactions.

**Availability and implementation:**

The GraphaRNA source code is available on GitHub (github.com/mjustynaPhD/GraphaRNA); training/test datasets and pre-trained model weights are provided on Zenodo (zenodo.org/records/13750967).

## 1 Introduction

Ribonucleic acid (RNA) is fundamental to a wide range of biological processes across all living organisms. It plays a critical role in gene transcription regulation, protein synthesis, and many other cellular functions. RNA also constitutes the genetic material of some pandemic-causing viruses, including HIV and SARS-CoV-2. In medicine, this molecule serves as a valuable biomarker for cancer detection and a target in cancer therapeutics (Xi *et al.* 2017, Zhu *et al.* 2022). Understanding the full spectrum of RNA functions is based heavily on structural studies, with a particular focus on deciphering the three-dimensional shape of this molecule.

For decades, researchers have studied 3D structures of RNA, initially relying on experiments such as X-ray crystallography and Nuclear Magnetic Resonance spectroscopy. However, accurate resolving of RNA structures through these techniques has proven challenging. Cryo-EM (Cryo-Electron Microscopy), which has recently gained popularity, also produces data with suboptimal resolutions, typically between 4 and 10 Å (Ma *et al.* 2022). Consequently, there is a pressing need for accurate *in silico* methods to predict RNA 3D structures. In the era of artificial intelligence (AI), the latter is expected to surpass traditional approaches, as seen in the protein domain, where AlphaFold (Senior *et al.* 2020, Jumper *et al.* 2021b) outperforms all competitors (AlQuraishi 2019, Jumper *et al.* 2021a). However, the RNA field lags behind, as shown in CASP15 (Das *et al.* 2023) and CASP16 (CASP16 2024, Kretsch *et al.* 2025), where the top prediction groups relied on cryo-EM data, heuristics, or molecular dynamics rather than AI.

Despite advances in deep learning (DL) for RNA structure prediction (Bernard *et al.* 2024), no model has yet consistently outperformed non-machine learning approaches across all RNA families. Methods such as RhoFold (Shen *et al.* 2022), trRosettaRNA (Wang *et al.* 2023), and AlphaFold3 (Abramson *et al.* 2024) have shown promising results for well-represented families but struggle to generalize to less-characterized RNAs. This limited generalization ability is largely due to the scarcity of available high-quality RNA structures for training and validation. As of March 2025, the Protein Data Bank (PDB) (Berman *et al.* 2000) contains over 200 000 protein structures but only 8466 RNA structures, with just 2584 remaining after filtering for non-redundant, high-resolution (<3.5 Å) entries. Additionally, RNA structural data are heavily skewed, with tRNA and rRNA making up 26% and 61% of known structures, respectively (Schneider *et al.* 2023). This uneven representation strongly affects the performance of current state-of-the-art methods, which typically rely on MSA-based transformer architectures that represent residues as frames and learn relative orientations between adjacent residues (RhoFold and trRosettaRNA), or use diffusion models to predict atomic coordinates directly [e.g., AlphaFold3, Boltz1 (Wohlwend *et al.* 2024)]. While this approach is effective for RNA families with abundant sequence homologs, its generalization drops significantly for less-represented families. Other methods, such as Ares (Townshend *et al.* 2021) and PAMNet (Zhang *et al.* 2022), take a different approach by employing Graph Neural Networks (GNNs) to predict the Root Mean Square Deviation (RMSD) of candidate structures generated by methods like FARFAR (Watkins *et al.* 2020). However, these models also face challenges in generalization, underscoring the need for approaches that can effectively model diverse RNA structures beyond well-characterized families (Hvidsten *et al.* 2009, Antczak *et al.* 2016).

Here, we explore the potential of GNNs as an alternative approach to RNA 3D structure prediction. These models have already proven effective in various biologically and chemically oriented tasks, including linker prediction (Igashov *et al.* 2024), molecular conformer generation (Xu *et al.* 2022), and docking pose prediction (Corso *et al.* 2022). We apply them to reconstruct RNA interactions at the atomic scale while employing a coarse-grained representation of the molecule structure. Our approach, implemented in the GraphaRNA system, relies on a generative framework based on the denoising diffusion probabilistic model (DDPM) (Rombach *et al.* 2022), which allows learning of atomic interactions and structural properties, such as torsion and bond angles, as well as interatomic distances. To achieve this, we adapted the PAMNet architecture (Zhang *et al.* 2023) and integrated it with an RNA language model (Penić *et al.* 2025) and a transformer module (Vaswani *et al.* 2023), enabling prediction of atomic positions under user-defined constraints. Unlike many existing approaches, GraphaRNA can incorporate a user-specified 2D structure, making it especially suitable for hypothesis-driven modeling based on partial experimental data—such as SHAPE reactivity, chemical probing, or mutational analysis. This flexibility supports the exploration of mutation effects, assessment of alternative pairing schemes, and testing of mechanistic hypotheses, offering a valuable complement to purely sequence-driven pipelines. For model training, we compiled a dataset of small RNA 3D substructures (local descriptors) (Antczak *et al.* 2016), which capture fine-grained residue-level interactions. This strategy enabled the construction of a sufficiently large training dataset. To evaluate generalization, we split the data by RNA family: training only on rRNA and tRNA descriptors, and testing on descriptors from all other families. Our results show that GraphaRNA can effectively predict atomic-resolution local 3D RNA descriptors while accommodating user-defined sequence and Watson–Crick–Franklin (WCF) interaction constraints.

## 2 Materials and methods

### 2.1 Local 3D RNA descriptors

**Figure 1.**
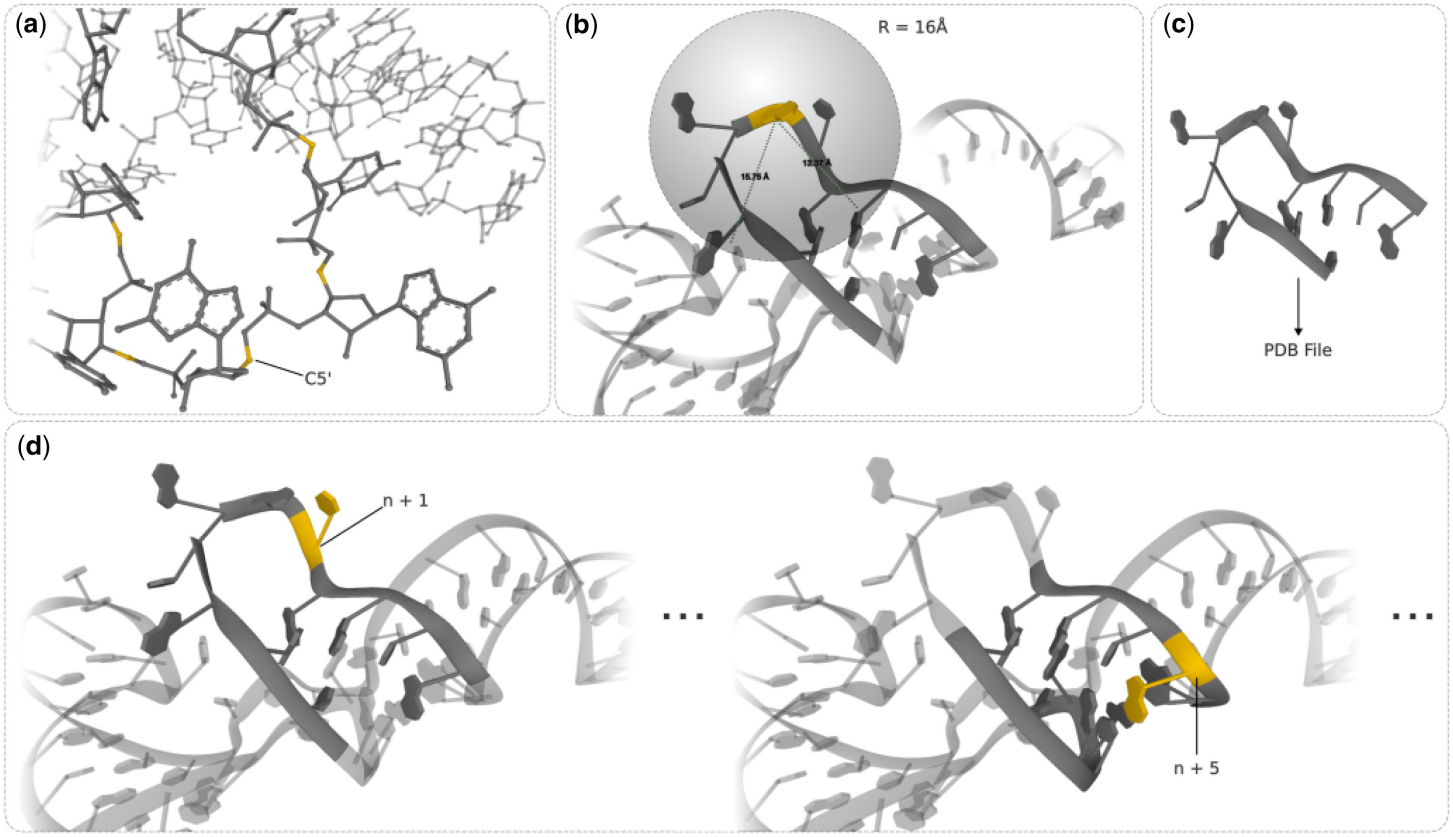
Extraction of local 3D RNA descriptors. (a) The C5${}^{\prime }$ atom is selected for each residue as the central reference point. (b) Distances are calculated between the central residue and all other residues; all residues within a 16 Å radius form a descriptor. (c) The descriptor is extended by adding two additional residues on each side of the both central and in-contact residue to ensure robustness; its final structure is saved in the PDB file. (d) Steps (a)–(c) are repeated iteratively for each residue in the RNA molecule considered.

Local 3D RNA descriptors (Antczak *et al.* 2016) are substructures extracted from the original RNA molecule, designed to encode the spatial neighborhood of each residue. The process of generating these descriptors involves *N–4* iterations, with *N* corresponding to the number of residues in the analyzed RNA (Fig. 1). The algorithm processes successive residues—starting with the 3rd and ending with (*N-2*)th residue—and performs 3 operations for each. In the first operation, the central atom of the current residue, in this case C5${}^{\prime }$, is selected. Next, all in-contact residues within a specified distance threshold *T* are identified and included in the descriptor; the distances are computed between the C5${}^{\prime }$ atoms. The descriptors must be large enough to capture relevant residue-residue interactions; however, it is important to note that increasing the threshold also increases the complexity of the descriptors, which in turn requires more computational power for model training. In this study, the threshold *T* was set to 16 Å. This value was determined based on the maximum distance observed between the coordinates of the $C5^{\prime }$ atoms of residues involved in canonical or non-canonical interactions within a non-redundant, high-resolution reference dataset. The descriptor may consist of one or more discontinuous strands, termed segments. These segments are extended in the third operation by adding two additional residues for every central and in-contact residue in the descriptor. This ensures that the segments are sufficiently large, comprising at least five residues. Additional residues are not required to be within the *T* threshold.

A notable feature of local 3D RNA descriptors is their recurrence in non-homologous structures, indicating that similar local environments can be found across diverse RNA molecules. This unique characteristic presents an opportunity to design models capable of generalizing to new RNA families. Depending on the size of the parent structure, the 3D arrangement of its strands, and the overall compactness, a single RNA molecule can generate multiple descriptors of varying sizes.

### 2.2 Training and testing sets

The training and test sets were constructed from local 3D RNA descriptors. We began by downloading non-redundant RNA representatives from RNAsolo (Adamczyk *et al.* 2022), selecting only structures with a resolution of 3.5 Å or better. The data set, time-stamped March 2023, comprised 1564 experimental structures. From these, we generated 177 629 local descriptors ranging from 1 to 18 segments using the descs-standalone package (Antczak *et al.* 2016). Next, we performed data cleaning to reduce redundancy and eliminate repetitive or uninformative substructures. Specifically, we excluded unfolded single-stranded fragments and all descriptors with more than 80% sequence identity or more than three segments, reducing the set to 76 067 descriptors. To further address structural redundancy, we performed spatial alignment using descs-standalone and applied additional filtering to ensure structural diversity. Two descriptors were considered redundant if they met a set of geometric similarity criteria inspired by established approaches in protein structure comparison (Hvidsten *et al.* 2009, Daniluk and Lesyng 2011). In particular, descriptors were treated as structurally similar if: (i) the RMSD between their central elements was ≤2.5 Å; (ii) at least one duplex, consisting of the central element and one additional segment, had an RMSD ≤4 Å; (iii) the alignment covered more than half of the structural elements and more than two-thirds of the residues; and (iv) the overall alignment RMSD was ≤3.5 Å. A modified Hungarian algorithm, allowing partial alignments, was used to perform descriptor matching. When multiple descriptors satisfied these criteria, only one representative was kept in the dataset, resulting in the removal of 31 966 redundant entries. The final dataset, named RNA3Desc, comprised 3636 one-segment descriptors, 16 849 two-segment descriptors, and 23 616 three-segment descriptors. We observed that each descriptor contained no more than 60 nucleotides, ensuring a controlled sequence length for training. Since DL models for RNA structure prediction are often biased toward well-represented RNA families, careful dataset partitioning is essential (Justyna *et al.* 2023). To mitigate this, we divided the collection according to Rfam family assignments (Kalvari *et al.* 2021): descriptors associated with rRNA or tRNA (36 449 entries) were assigned to the training set, while all others (7652 structures) were used for testing. Table S2, available as supplementary data at *Bioinformatics* online summarizes Rfam families represented in the test set, and Fig. S1, available as supplementary data at *Bioinformatics* online shows the distribution of descriptor sizes.

### 2.3 Model implementation and training

**Figure 2.**
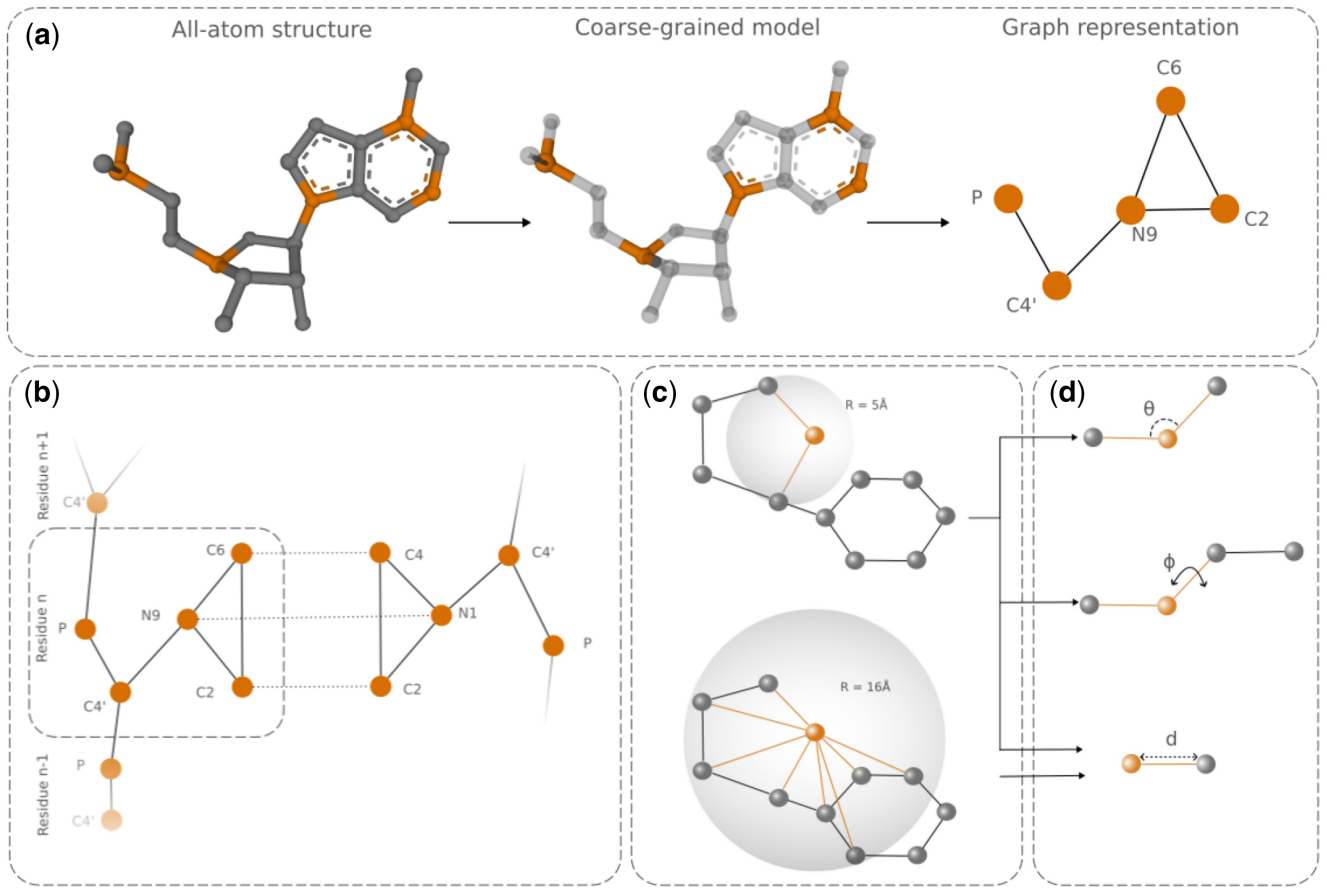
Graph construction. (a) Transformation from all-atom via coarse-grained to graph representation illustrated for purine. Orange represents coarse-grained atoms, while light gray (middle panel) denotes elements excluded from the coarse-grained model. (b) Each node in the graph represents an atom. Solid edges denote covalent bonds between atoms, while dotted edges represent interactions derived from user-provided 2D structure constraints. This dual-edge system enables the model to capture both the structural connectivity and the additional interactions specified in the input. (c) Close (top) and long-range (bottom) interactions between neighboring atoms. (d) Feature computation between nodes. For close interaction, we compute the distance d and two types of angles—θ between the node and the neighbors in one hop and ϕ between the node and neighbors in two hops. For a long-range interaction, we only compute the distances.

GraphaRNA was implemented in PyTorch (version 2.3.0), with graph components built using PyTorch Geometric (PyG, version 2.5.3). The connectivity graph was constructed in COO format, and nodes represented atoms in the coarse-grained model. Node features were one-hot encoded to capture atom type (carbon, phosphorus, or nitrogen), a C4${}^{\prime }$ flag, and residue type (A, G, C, or U). Additionally, nodes stored vector-based information for sequence embeddings from the RNA language model (size of 256) and time embeddings (size of 16) for the denoising steps. Due to memory constraints and the need for numerical stability, we limited GraphaRNA to six blocks of PAMNet. The atomic coordinates were processed through a linear layer with layer normalization (Ba *et al.* 2016) and sigmoid activation, encoding them as 256-dimensional vectors. The graph nodes were connected in a stepwise process, beginning with intraresidue contacts (covalent bonds), followed by interresidue interactions defined by the user-provided 2D structure (Fig. 2). Technically, the input can be extended to include non-canonical interactions; however, this remains challenging in practice due to the inconsistencies among current base-pairing annotation methods. The interaction types were encoded as edge features. Edge attributes included information about angles (for edges that represent close contacts) and distances (for edges that represent long-range interactions). In the case of close contacts, features were computed based on angles, including both one-hop and two-hop neighbors (the latter similar to torsion angles), as shown in Fig. 2. Angle information was processed using spherical (7 harmonics) and radial (16 parameters) basis functions (Gasteiger *et al.* 2020) and then passed through a trainable linear layer to generate embeddings. In addition, new edges were dynamically added to the graph based on defined distance thresholds: 5 Å for close contacts and 16 Å for long-range interactions. This implies that, in theory, the maximal distance over which signals can propagate in the GNN is approximately 30 Å for local interactions and 96 Å for long-range ones. To improve computational efficiency, each node was connected to only its 20 nearest neighbors, meaning the actual signal propagation distances may vary depending on the atomic spatial distribution.

**Figure 3.**
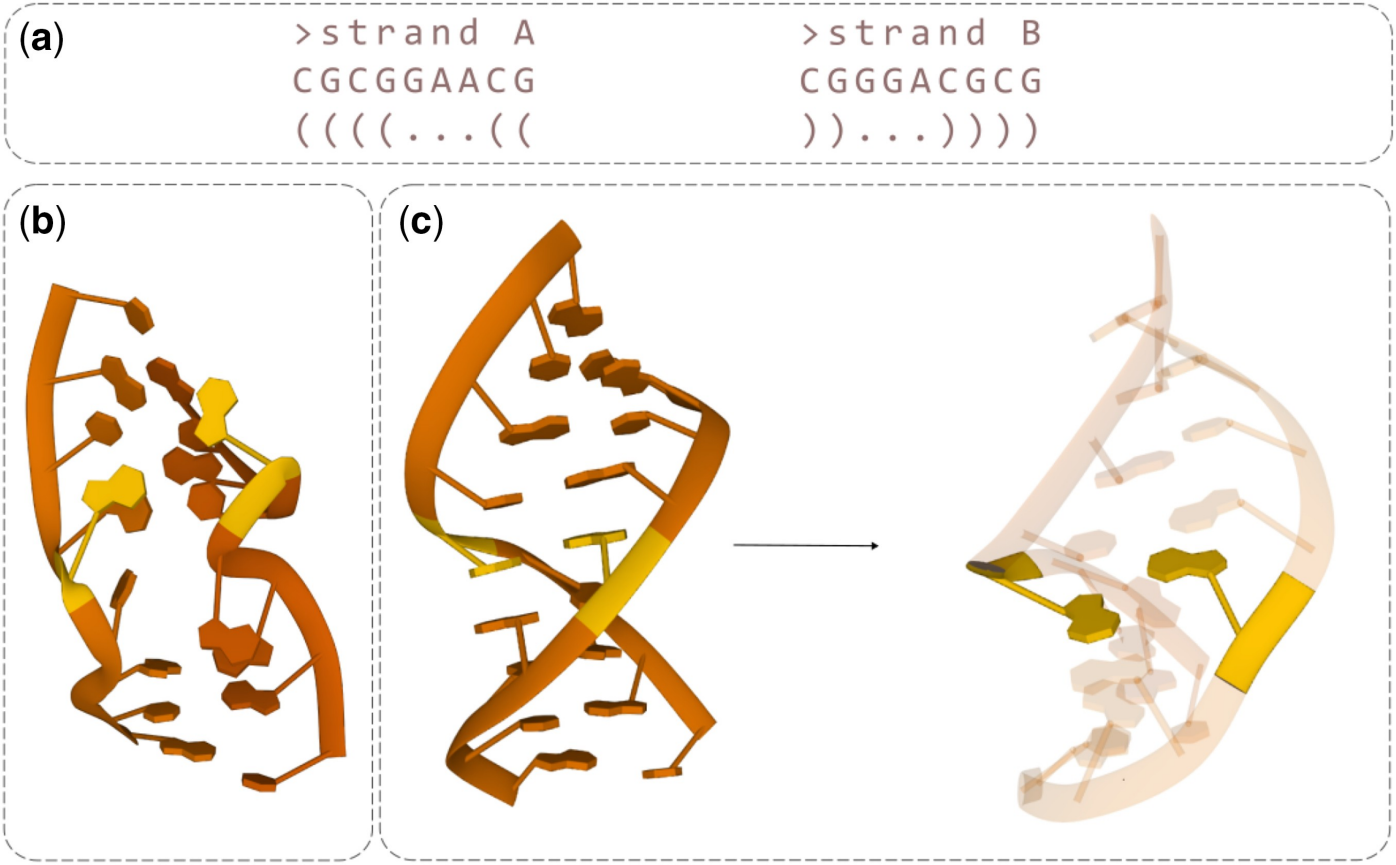
An example of the impact of RNA language embeddings on model predictions. (a) The input sequence and 2D structure. (b) A predicted helix with an expected non-canonical tSH base pair in the middle. Since this base pair is not explicitly defined in the user input, it is represented as a dot (“.”). The model learns to maintain canonical base pairs, while non-canonical ones tend to be pushed apart. (c) The positive effect of embeddings: the predicted structure retains its helical shape, and instead of being displaced, the residues adopt an orientation that enables the formation of the expected tSH base pair. The yellow color presents an example of the tSH base pair in the corresponding structures.

For the RNA language component, we used the RiNALMo model with the giga-v1 weights (Penić *et al.* 2025). Although other RNA language models (RNA-LM) exist, such as RNA-FM (Chen *et al.* 2022), RiNALMo was the largest available at the time of implementation. It had also demonstrated strong performance in multiple downstream tasks and benefited from FlashAttention (Dao *et al.* 2022), which enhanced computational efficiency of the model. During training, we kept RiNALMo frozen and introduced a linear layer (without bias) containing 1280 neurons with ReLU activation (Agarap 2019). The output embedding size was 256. In cases involving multiple RNA segments, their sequences were merged into a single string to maintain sequential context and improve computational efficiency, rather than processing each segment independently. The RNA language model generated meaningful nucleotide embeddings, which were subsequently used as features in the GNN. A single embedding was produced for each nucleotide and assigned as a feature to all five atoms representing that nucleotide in the model. As illustrated in Fig. 3, these embeddings positively influenced the predictions by capturing essential sequence context. Notably, we observed that the RNA-LM introduces an inductive bias based solely on sequence information, guiding the model toward the correct 3D structural conformation. In contrast, one-hot encoding alone provides no meaningful insight into nucleotide relationships. With RNA-LM embeddings, the model can recognize and learn common RNA structure patterns, such as non-canonical pairings, hairpin loops, and others. Traditionally, MSA has been the gold standard for many structure prediction methods, including trRosettaRNA, RhoFold, AlphaFold3, and DeepFoldRNA. However, MSA-based approaches come with significant challenges: running them locally requires extensive storage (up to 2TB of disk space), and the process itself can be highly time-consuming. RNA language models offer a promising alternative, addressing these limitations while effectively capturing sequence-derived structural features. Table S2, available as supplementary data at *Bioinformatics* online presents an ablation study assessing the impact of the RNA language model by comparing it with a simpler, sequence-based one-hot encoding.

The training process considered at most three segments; however, this is not a strict constraint of the method, allowing generating predictions for a larger number of strands. During training, we employed 5000 denoising steps with a linear noise scheduler. The training process ran for 800 epochs with a batch size of 128, taking ∼40 hours. The model was trained in a distributed manner using 8 Nvidia A100 GPUs. To enhance numerical stability and prevent exploding gradients, we applied gradient clipping with a threshold of 2. Additionally, a step learning rate scheduler was used, adjusting the initial learning rate of 0.003 by a factor of 0.9 every 30 epochs. Additional details on the model implementation are provided in the Supplementary Materials (Section Implementation Details and Table S1, available as supplementary data at *Bioinformatics* online).

### 2.4 Model evaluation

The RNA 3D structure predictions were evaluated using three common metrics: RMSD, eRMSD (epsilon RMSD) (Bottaro *et al.* 2014), and INF (Interaction Network Fidelity) (Parisien *et al.* 2009). RMSD is a general distance measure that is used to assess the structural accuracy of biological molecules, whereas eRMSD (a distance measure) and INF (a similarity measure) are specifically tailored for the evaluation of RNA structures.

RMSD was calculated with the open-source PyMOL tool (version 3.0.0). Since RMSD operates on full-atom structures, the predicted coarse-grained models were converted into full-atom representations with ARENA (Perry *et al.* 2023). RMSD was then calculated across the entire structure using Equation 1, where *N* is the total number of atoms, *r*^pred^ represents the predicted atomic coordinates, and *r*^true^ denotes the corresponding coordinates in the ground truth structure.

(1) $$\text{RMSD}=\sqrt{\frac{1}{N}\sum_{i=1}^{N}{\left(r_{i}^{\text{pred}}-r_{i}^{\text{true}}\right)}^{2}}$$

eRMSD, which also requires a full-atom structure, was calculated using Barnaba (Bottaro *et al.* 2019). This metric evaluates the relative arrangement of nucleobases and their distances, making it particularly sensitive to base pairing. eRMSD is continuous and symmetric, with typical values interpreted as follows: values below 0.7 indicate native-like stems and loops, values between 0.7 and 1.3 correspond to structures with native stems but non-native loops, and values greater than 1.3 are indicative of significant deviations from the native state. As defined in (2), eRMSD quantifies differences in base-base interactions by applying a nonlinear function *G* to the pairwise distances derived from rescaled position vectors ${\tilde{r}}_{ij}$ of selected base atoms.

(2) $$\epsilon \text{RMSD}=\sqrt{\frac{1}{N}\sum_{i,j}{\left(G({\tilde{r}}_{ij}^{\text{pred}})-G({\tilde{r}}_{ij}^{\text{true}})\right)}^{2}}$$

INF evaluates discrepancies between the predicted and reference secondary structures, providing a normalized score between 0 and 1. To compute this, we first extracted base pair information from both the predicted and reference structures using the RNApolis package (M. Szachuniuk 2019). INF was then calculated for canonical base pairs according to (3) (Parisien *et al.* 2009). True positives (TP) represent interactions that are present in both the predicted and ground truth structures. False positives (FP) refer to interactions predicted by the model but absent in the ground truth, while false negatives (FN) are interactions present in the ground truth but missing from the prediction.

(3) $$\text{INF}=\sqrt{\frac{\left|\text{TP}\right|}{\left|\text{TP}|+|\text{FP}\right|}\times \frac{\left|\text{TP}\right|}{\left|\text{TP}|+|\text{FN}\right|}}$$

## 3 Results

### 3.1 Neural network model

**Figure 4.**
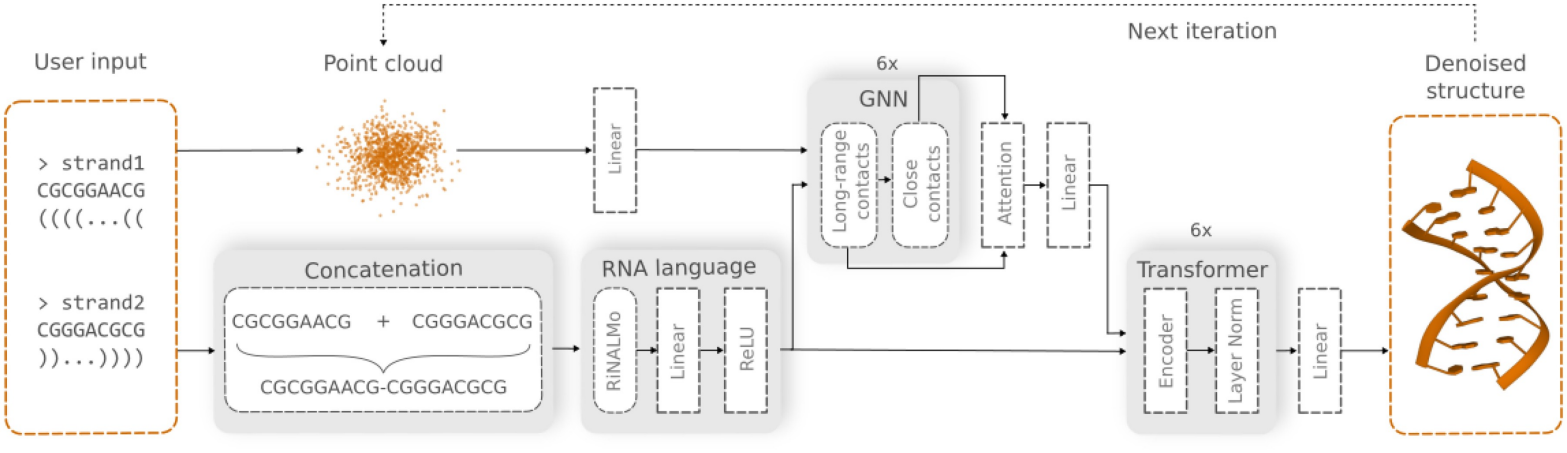
Components of the GraphaRNA model. The user input is provided in the Vienna text format, defining the sequence and the expected secondary structure. These are used to construct the static graph. Atom positions are initially represented as a random point cloud and are iteratively denoised by the diffusion model.

GraphaRNA (GNN and diffusion model for RNA 3D structure prediction) comprises three major components: an RNA language model, a GNN, and a transformer (Vaswani *et al.* 2023) (Fig. 4). The model takes a dot-bracket-like input provided by the user, defining the RNA sequence and the secondary structure. GraphaRNA then predicts the 3D structure while ensuring that all specified 2D contacts are preserved.

Inspired by a flow-matching approach (Lipman *et al.* 2023, Huguet *et al.* 2024) to protein design that incorporates a protein language model (Lin *et al.* 2022), we applied a similar concept to RNA. For this, we used RiNALMo (Penić *et al.* 2025), an RNA language model pre-trained on all RNA sequences from RNAcentral (Petrov *et al.* 2017), which provides a strong inductive bias and significantly enhances the performance of GraphaRNA. When dealing with multi-segment descriptors, concatenation is necessary to run the RNA language model and generate meaningful embeddings for all residues. This process allows the model to capture the context of all segments. While one might argue that this is not the ideal approach—since the embedding should ideally be generated for the entire sequence—this method is often required in cases where the sequence of a particular fragment is undefined or for duplexes.

As the second component, we chose a GNN, currently considered a state-of-the-art approach in molecular modeling (Townshend *et al.* 2021, Huguet *et al.* 2024, Igashov *et al.* 2024, Joshi *et al.* 2024). We wanted it to consider close contacts (interactions between atoms 0–5 Å apart) and long-range interactions (up to 16 Å) that are critical for predicting RNA 3D structure. Thus, we used PAMNet (Zhang *et al.* 2023) and adjusted it to accommodate both types of interactions, with each of its two layers responsible for handling a specific interaction type. Another modification enabled PAMNet to return a vector containing the coordinates and types of atoms, and the types of residues, instead of a single value. This was achieved by removing the global pooling operation from the final layer. The input dimensions were adjusted to include features such as input coordinates, one-hot encoded atom types, residue embeddings, and denoising time steps. PAMNet uses a global message-passing mechanism in which information about each atom (node) is passed to its neighbor atoms in a single run.

The transformer encoder architecture was inspired by Invariant Point Attention (IPA), originally implemented in AlphaFold (Jumper *et al.* 2021b). It consists of six layers with eight attention heads. Incorporating this component significantly improved the quality of the RNA structures compared to using the GNN alone. GNN efficiently feeds the transformer with structural embeddings.

Although the overall architecture may seem complex, each component plays a meaningful role in enhancing prediction quality. To assess the contribution of individual elements, we conducted ablation studies, as summarized in Table S2, available as supplementary data at *Bioinformatics* online. Specifically, we evaluated the impact of the RNA language model, as well as the number of GNN and transformer layers, on overall model performance. The evaluated model variants are described in detail in Table S3, available as supplementary data at *Bioinformatics* online.

### 3.2 Training

Due to memory constraints and the need for computational efficiency, we adopted a coarse-grained 5-atom representation of the RNA structure, as proposed by SimRNA (Boniecki *et al.* 2016). This representation offers a balance between all-atom and simplified models, reducing computational cost by limiting the atom set while preserving essential geometric details, including base pair orientation and edge-specific interactions such as sugar and Hoogsteen faces. Each residue is represented by two backbone atoms (P and C4${}^{\prime }$) and three nucleobase atoms (N1-C2-C4 for pyrimidines, N9-C2-C6 for purines). During data preprocessing, we converted each full-atom structure into a coarse-grained model and constructed the corresponding graph of interatomic interactions (Fig. 2a). This graph was further supplemented with edges representing canonical base-pair interactions, which were added based on the extracted secondary structure (Fig. 2b). Static edges were used to represent covalent bonds between atoms or interactions between canonical base pairs, remaining fixed regardless of atomic distances. In contrast, dynamic edges were created between pairs of atoms that did not have an existing edge but were within a defined distance threshold. This approach ensures that two residues remain close in the RNA structure, even when their atoms are spatially distant in the early stages of the prediction process. To maintain consistency across all structures, we centered them at the point (0,0,0) by computing and subtracting the mean of the original coordinates. Finally, for numerical stability, we scaled the coordinate values by a factor of 10.

Each RNA structure in the set was gradually corrupted through a diffusion process using the standard DDPM framework (Ho *et al.* 2020). It involved adding Gaussian noise to the atomic coordinates in multiple steps while preserving atomic properties (such as atom names) and the connectivity graph. The neural network model of GraphaRNA was trained to reverse this process by predicting a less noisy (previous) state at each step. This approach enabled the model to learn to reconstruct the original 3D structure from noisy input data.

During inference, we first create a graph representing the RNA structure to be predicted. This graph includes all molecular features (e.g., atom types, residue embeddings) and the topology derived from the input sequence and 2D interactions. The missing information—atomic coordinates—is initialized as random noise. The model then performs denoising steps on these coordinates, gradually refining them with each iteration. At the end of the denoising process, the final structure is determined.

### 3.3 Evaluation

We benchmarked GraphaRNA against Boltz1 (Wohlwend *et al.* 2024), RhoFold (Shen *et al.* 2024), and DRFold (Li *et al.* 2023) by comparing predicted RNA 3D structures to ground truth data. Four metrics—RMSD (Kabsch 1976), eRMSD (Bottaro *et al.* 2014), INF (Parisien *et al.* 2009), and lDDT (Mariani *et al.* 2013)—were used for evaluation. INF was measured separately for canonical (Watson–Crick–Franklin) base pairs as INF(WCF), and for non-canonical base pairs as INF(NWC). Boltz1 was chosen for comparison because, like GraphaRNA, it is a generative model that supports user-defined multi-stranded inputs. Moreover, Boltz1 provides competitive prediction quality compared to AF3 and is straightforward to install on a cluster. RhoFold, though not generative, incorporates multiple sequence alignments during prediction, whereas DRFold, an *ab initio* method, relies on PETfold (Seemann *et al.* 2008) and RNAfold (Lorenz *et al.* 2011) for 2D structure prediction without MSA. Unlike these methods, GraphaRNA requires the user to provide a 2D structure. For this reason, the INF(WCF) metric for GraphaRNA reflects its ability to preserve predefined constraints rather than predict an accurate 2D structure (Fig. 6).

**Figure 5.**
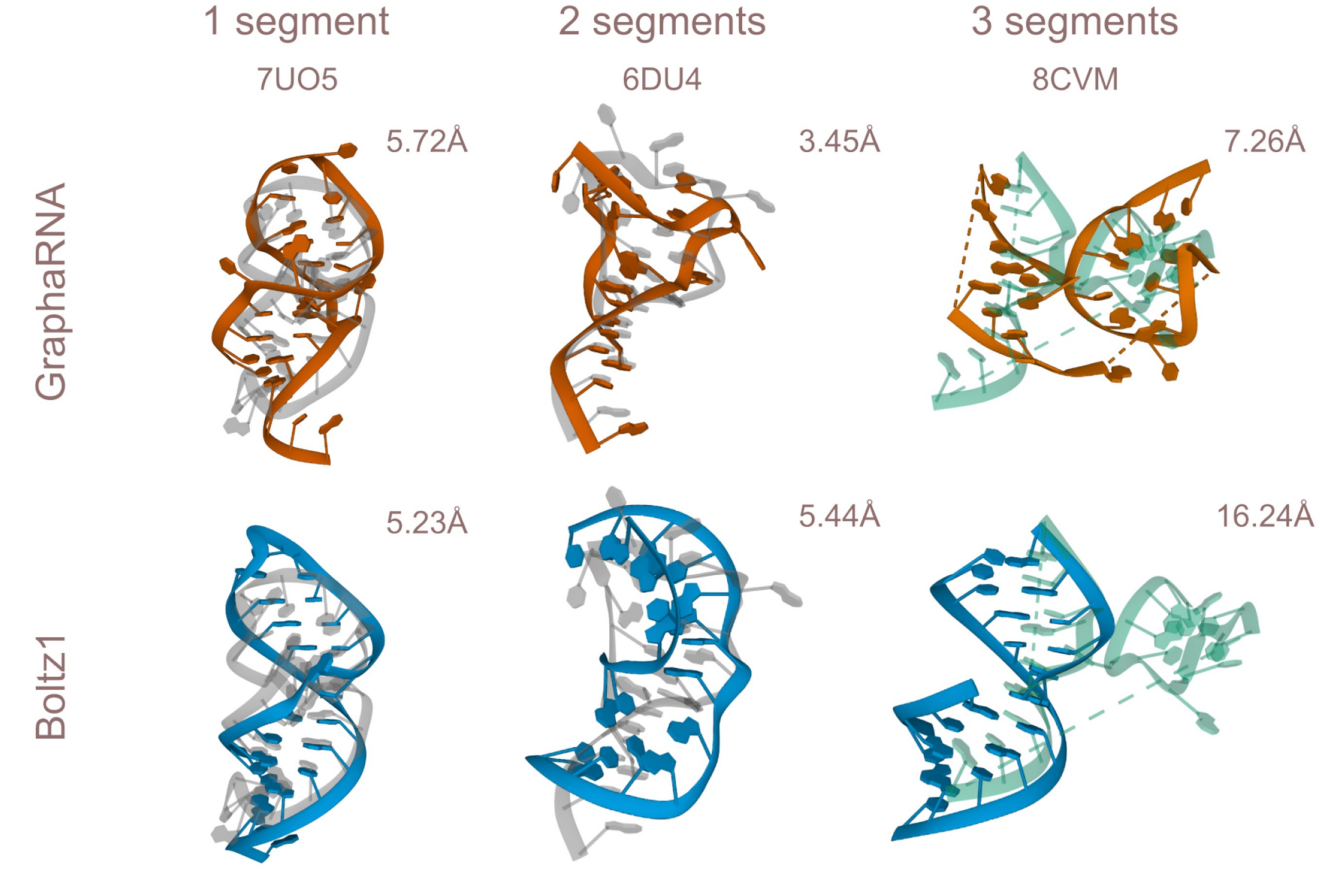
Example descriptors extracted from ground truth structures (7UO5, 6DU4, and 8CVM) in gray, aligned with their predictions by GraphaRNA (orange) and Boltz1 (blue). For 1- and 2-segment prediction, both GraphaRNA and Boltz1 can generate competitive structures. For 3 segments (and more), the problem is not only a proper structure folding but also the spatial arrangement of the components.

We evaluated all tools using the full set of RNA descriptors from the test set, with results compared to ground truth data shown in Table 1, which categorizes performance by RNA descriptor type. Each prediction was generated using the sequences corresponding to the individual descriptor segments. Although GraphaRNA was trained on descriptors with 1–3 segments, we also tested its generalization to 4 and 5 segments. Single-segment descriptors, consisting of one sequence, are compatible with methods like RhoFold and DRFold. As shown in Table 1, RMSD increases as the number of segments grows. For 1- and 2-segment descriptors, the primary challenge is generating the correct fold of the structure. For 3 or more segments, even if the fold of each component is accurate, its spatial arrangement can differ, resulting in higher RMSD (see Fig. 5). Lastly, due to the nature of the descriptors, our model is more attuned to external contacts, whereas Boltz1, trained on full RNA 3D structures, tends to produce more helical shapes.

**Figure 6.**
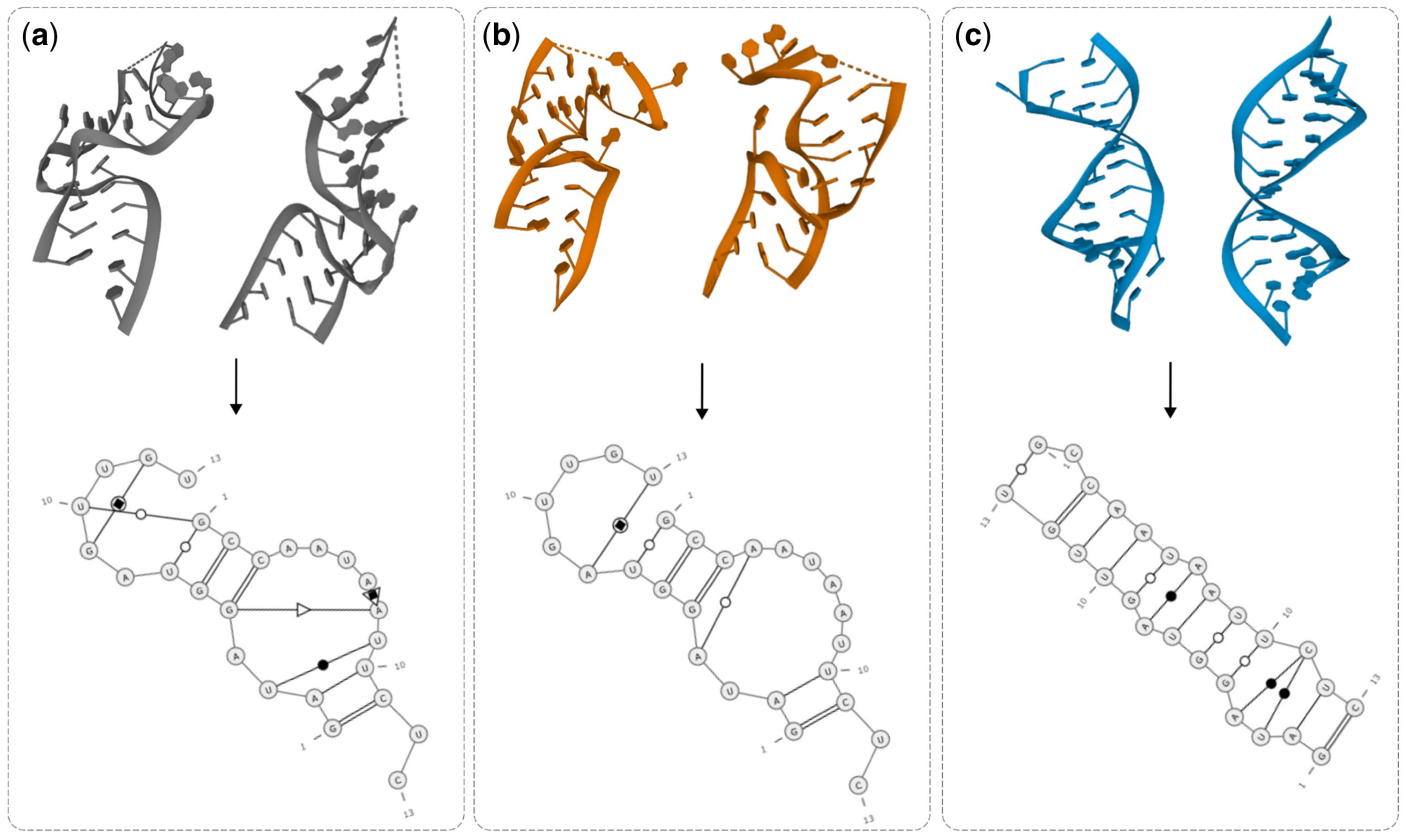
Example of a 2-segment descriptor in (a) the ground-truth structure (PDB ID: 6YW5), and predictions by (b) GraphaRNA and (c) Boltz1. GraphaRNA preserves user-defined constraints (WCF contacts) during inference and introduces some non-canonical base pairs, though these are not explicitly specified by the user. Boltz1, on the other hand, determines the 2D structure using MSA, resulting in deviations from the ground-truth configuration.

**Table 1.** Mean and SD of RMSD, eRMSD, INF(WCF), INF(NWC), and lDDT for each descriptor group.

Method	RMSD [Å] ↓	eRMSD ↓	INF(WCF) ↑	INF(NWC) ↑	lDDT ↑
	1-segment descriptors (1245 samples of length 8–34 nt)				
GraphaRNA	3.36 ± 2.52	1.27 ± 0.29	**0.97** ± **0.12**	0.24 ± 0.31	0.67 ± 0.11
Boltz1	**3.28** ± **3.17**	**1.25** ± **0.47**	0.78 ± 0.33	**0.38** ± **0.37**	**0.71** ± **0.15**
RhoFold	6.06 ± 4.44	1.40 ± 0.29	0.44 ± 0.46	0.12 ± 0.26	0.55 ± 0.14
DRFold	6.15 ± 4.29	1.42 ± 0.31	0.28 ± 0.42	0.20 ± 0.27	0.57 ± 0.16
	2-segment descriptors (3752 samples of length 12–42 nt)				
GraphaRNA	6.65 ± 3.18	**1.43** ± **0.26**	**0.96** ± **0.10**	0.19 ± 0.25	**0.51** ± **0.16**
Boltz1	**6.51** ± **4.36**	1.51 ± 0.44	0.70 ± 0.27	**0.23** ± **0.31**	0.36 ± 0.19
RhoFold	–	–	–	–	–
DRFold	–	–	–	–	–
	3-segment descriptors (2655 samples of length 18–58 nt)				
GraphaRNA	9.66 ± 2.89	**1.56** ± **0.21**	**0.96** ± **0.09**	0.19 ± 0.20	**0.47** ± **0.11**
Boltz1	**9.60** ± **4.41**	1.73 ± 0.36	0.65 ± 0.22	**0.24** ± **0.25**	0.20 ± 0.10
RhoFold	–	–	–	–	–
DRFold	–	–	–	–	–
	4-segment descriptors (2959 samples of length 25–60 nt)				
GraphaRNA	**11.65** ± **2.94**	**1.56** ± **0.16**	**0.82** ± **0.16**	0.12 ± 0.17	**0.39** ± **0.09**
Boltz1	12.33 ± 4.19	1.79 ± 0.32	0.61 ± 0.20	**0.21** ± **0.22**	0.14 ± 0.06
RhoFold	–	–	–	–	–
DRFold	–	–	–	–	–
	5-segment descriptors (1376 samples of length 34–62 nt)				
GraphaRNA	**13.25** ± **2.70**	**1.60** ± **0.15**	**0.81** ± **0.16**	0.13 ± 0.16	**0.36** ± **0.08**
Boltz1	14.97 ± 3.40	1.86 ± 0.26	0.57 ± 0.19	**0.20** ± **0.20**	0.11 ± 0.04
RhoFold	–	–	–	–	–
DRFold	–	–	–	–	–

Arrows in the column headers indicate the interpretation of each metric: ↑ denotes a similarity measure (higher values indicate more similar structures), while ↓ denotes a distance measure (lower values indicate more similar structures). Best values in each group are highlighted in bold.

Evaluation of all models was performed using the versions released by their respective authors. While this approach may introduce some bias—since structures from our test set could have been seen during training—it reflects a realistic usage scenario. Ideally, a fully fair comparison would involve retraining all models on the same dataset, but this was not feasible due to computational limitations.

## 4 Conclusions

In this study, we introduce GraphaRNA, a method that captures interatomic interactions and predicts the 3D shapes of small RNA substructures. Unlike existing predictive tools, which primarily rely on MSA and pairwise distances, our approach applies GNNs to provide a more physics-aware representation of 3D RNA structure. While GNNs have previously been used to assess model quality by estimating RMSD, their potential for directly predicting atomic positions has remained largely unexplored. GraphaRNA addresses this by integrating a diffusion model with GNN-based processing, enabling direct generation of 3D RNA conformations.

Although we achieved competitive performance in predicting local 3D RNA descriptors, extending this success to entire RNA structures, particularly larger ones, remains challenging due to memory limitations and computational costs. Our method can handle structures of moderate size (≤100 nucleotides), but larger RNAs require reducing the number of layers and interactions with neighboring atoms to fit within GPU memory, which impacts performance. The accuracy of the model depends on the number of interatomic edges considered; however, increasing them significantly raises memory usage and computation time, limiting scalability. Future research could address this by optimizing the selection of dynamic edges or adopting an alternative coarse-grained representation, such as a 3-atom model. Additionally, the current approach predicts the coordinates of each atom individually, and incorporating an equivariant representation could enhance the structural accuracy and overall performance of the model.

A limitation of the current approach is that RNA-LM embeddings are generated from concatenated descriptor segments. Computing embeddings from the full RNA chain and then extracting the relevant positions might better capture contextual information. Addressing this limitation could be a focus of future work. Another consideration is the potential bias introduced by using an RNA-LM such as RiNALMo, which was trained on all sequences from Rfam and RNACentral—making it likely that some test sequences were present in its training data. Future research could explore alternative representations for nucleotide embeddings or consider different RNA-LM architectures. An additional direction involves adapting the generative model for structure in-painting, enabling the targeted generation of structural elements, such as multi-loops, within a specified context.

While maximizing prediction accuracy is often the primary objective in structure prediction benchmarks, it is equally important to explore diverse methodological strategies that offer new capabilities and perspectives. GraphaRNA represents such an alternative: a distinct modeling paradigm that combines interatomic graph reasoning with generative diffusion processes. Although it may not consistently outperform highly optimized, ensemble-based pipelines, its unique architecture enables context-aware structure generation and provides complementary strengths that encourage innovation. In addition to predicting local RNA substructures, GraphaRNA offers a flexible framework for modeling multimeric assemblies and generating complex interactions, such as RNA-ligand or RNA-protein interfaces. Its generative nature supports structure refinement tasks by enabling targeted regeneration of specific structural fragments, including disordered or entangled regions. Future extensions—such as incorporating flow-matching techniques, adapting the model for in-painting, or integrating alternative coarse-grained representations—may further enhance its applicability. Methodological diversity reduces over-reliance on a single dominant approach and fosters new directions in RNA structural biology. We believe that combining GNNs with diffusion models creates a powerful and extensible foundation for addressing a wide range of problems, including RNA structure prediction, refinement, interaction modeling, and design.

## Supplementary Material

btaf515_Supplementary_Data

## Data Availability

The training and test datasets used in this work, along with the pre-trained model weights, are available for download from Zenodo (https://zenodo.org/records/13750967). The source code of GraphaRNA is publicly available at GitHub (https://github.com/mjustynaPhD/GraphaRNA).
