# Supplementary material for: Graph neural network and diffusion model for modeling RNA interatomic interactions
Source: Bioinformatics. 2025 Sep 15;41(9):btaf515. doi: 10.1093/bioinformatics/btaf515 (PMC12472125; doi:10.1093/bioinformatics/btaf515)
Supplement: btaf515_Supplementary_Data [file btaf515_supplementary_data.pdf]

Graph Neural Network and Diffusion Model for Modeling RNA Interatomic Interactions

Marek Justyna, Craig Zirbel, Maciej Antczak, Marta Szachniuk

Supplementary Materials

Contents

1 Implementation details **2**

2 Ablation studies **3**

3 Test set analysis **4**

3.1 Distribution of Rfam families 4

3.2 Distribution of test set descriptors across segment groups 6

1 Implementation details

GraphaRNA uses a coarse-grained model to represent RNA 3D structure, as proposed by SimRNA [1]. Each nucleotide in this 5-atom representation is modeled by five atoms: the phosphate (P), the sugar atom C4', and three nucleobase atoms (N1-C2-C4 for pyrimidines, N9-C2-C6 for purines). The feature vector for each atom includes:

- 3D coordinates (x, y, z),
- One-hot encoded atom types (C, N, P; 3 dimensions),
- One-hot encoded residue types (A, C, G, U; 4 dimensions),
- Binary flags for atom roles (C4', C2, C4/C6, N1/N9; 4 dimensions),
- RNA-LM (RiNALMo) embedding (256 dimensions),
- Time embedding for the diffusion process (16 dimensions).

In the diffusion process, the time embedding has 16 dimensions, with each integer timestep encoded using sinusoidal positional embeddings [4]. Specifically, the timestep t is transformed into a 16-dimensional vector using sine and cosine functions at varying frequencies, following the method of Vaswani *et al.* [4]. This embedding is then passed through two multilayer perceptron (MLP) layers with GELU activation functions to enhance trainability and adapt to the model's architecture. The resulting time embedding is concatenated with the atom feature vector.

The GraphaRNA model was trained using the Smooth L1 loss function [2], which offers a balance between robustness to outliers and sensitivity to small errors, compared to the standard mean squared error (MSE). It is defined as:

$$\mathcal{L}_{\text{SmoothL1}} = \frac{1}{N} \sum_{i=1}^{N} \begin{cases} 0.5(\hat{\epsilon}_i - \epsilon_i)^2/\beta, & \text{if } \|\hat{\epsilon}_i - \epsilon_i\| < \beta, \\ \|\hat{\epsilon}_i - \epsilon_i\| - 0.5\beta, & \text{otherwise}, \end{cases} \tag{1}$$

where $\hat{\epsilon}_i$ and ϵ_i are the predicted and true noise vectors for feature i, N is the number of features, and $\beta = 1.0$ controls the transition between mean absolute error (MAE) and mean squared error (MSE) loss.

For coordinate diffusion, we adapted an inpainting technique inspired by Rombach *et al.* [3]. During the diffusion process, Gaussian noise is added to the entire feature vector – excluding the time embedding – in accordance with Equation 2:

$$\mathbf{x}_t = \sqrt{\alpha_t}\mathbf{x}_0 + \sqrt{1 - \alpha_t}\epsilon, \quad \epsilon \sim \mathcal{N}(0, \mathbf{I}), \tag{2}$$

where $\mathbf{x}_0$ is the original feature vector, $\mathbf{x}_t$ is the noisy vector at timestep t, α_t is a time-dependent noise, and ϵ is Gaussian noise. The model predicts the noise ϵ, and during generation, only the coordinate components (x, y, z) are denoised, keeping atom types, residue types, and RNA-LM embeddings fixed. This ensures the user-defined sequence remains unchanged, preventing the model from altering atom or residue identities. A linear noise schedule was used for sampling over 5,000 timesteps.

Table S1: Hyperparameters used for training the GraphaRNA model.

**Parameter**	**Value**
Learning rate	0.001
Batch size (per GPU)	16
Number of epochs	800
Optimizer	Adam
Step LR scheduler	$\gamma = 0.9$, every 30 epochs
Diffusion timesteps	5000
Noise schedule	Linear
Smooth L1 β	1.0
Gradient clipping norm	2.0

2 Ablation studies

Ablation studies are used to evaluate the contribution of individual model components by systematically removing or modifying them and observing the effect on performance. In our case, we conducted ablation experiments to assess the importance of key architectural elements in the GraphaRNA model.

Table S2 presents results comparing the baseline model – consisting of 6 GNN layers, 6 transformer blocks, and RNA language model embeddings – with four alternative variants. The architectural details of these variants are summarized in Table S3. The variants are characterized as follows:

- Variant V1 disables the RNA language model and replaces it with a standard one-hot encoding of the nucleotide sequence.
- Variant V2 reduces the number of GNN layers from 6 to 3, while keeping the transformer stack unchanged.
- Variant V3 retains all 6 GNN layers but reduces the number of Transformer blocks from 6 to 3.
- Variant V4 uses a minimal GNN architecture with only 1 GNN layer and a deeper Transformer stack of 12 layers.

Ablation experiments were designed to assess the contributions of sequence embeddings, GNN depth, and transformer depth to overall model accuracy. The results, summarized in Table S2, demonstrate that both the RNA language model and a balanced architectural depth across the GNN and Transformer components are important for achieving optimal performance.

Table S2: Ablation study evaluating the impact of architectural choices and input embeddings. Arrows indicate whether higher (↑) or lower (↓) values are better. Metrics are reported as mean ± standard deviation. The best values are highlighted in bold.

Model Variant	RMSD [Å] ↓	eRMSD ↓	INF ↑	lDDT ↑
		**1 Segment**		
Baseline	**3.36 ± 2.52**	**1.27 ± 0.29**	**0.97 ± 0.12**	**0.67 ± 0.11**
V1	8.55 ± 2.40	1.97 ± 0.16	0.07 ± 0.17	0.39 ± 0.08
V2	5.61 ± 2.26	1.88 ± 0.16	0.11 ± 0.21	0.51 ± 0.09
V3	9.12 ± 1.71	2.21 ± 0.35	0.09 ± 0.20	0.34 ± 0.06
V4	7.65 ± 1.92	2.01 ± 0.22	0.08 ± 0.19	0.46 ± 0.07
		**2 Segments**		
Baseline	**6.65 ± 3.18**	**1.43 ± 0.26**	**0.96 ± 0.10**	**0.51 ± 0.16**
V1	11.11 ± 2.46	2.02 ± 0.18	0.07 ± 0.17	0.30 ± 0.09
V2	8.61 ± 2.88	2.20 ± 0.74	0.13 ± 0.21	0.39 ± 0.13
V3	11.10 ± 1.47	2.53 ± 0.37	0.11 ± 0.20	0.28 ± 0.09
V4	9.79 ± 1.87	2.16 ± 0.25	0.09 ± 0.18	0.37 ± 0.11
		**3 Segments**		
Baseline	**9.66 ± 2.89**	**1.56 ± 0.21**	**0.96 ± 0.09**	**0.47 ± 0.11**
V1	13.29 ± 2.26	2.08 ± 0.16	0.10 ± 0.17	0.28 ± 0.07
V2	10.86 ± 2.88	2.16 ± 0.81	0.14 ± 0.20	0.35 ± 0.09
V3	12.65 ± 1.32	2.90 ± 0.36	0.15 ± 0.19	0.28 ± 0.07
V4	11.69 ± 1.76	2.31 ± 0.31	0.11 ± 0.18	0.35 ± 0.08

Table S3: Variants of GraphaRNA predictive model tested in the ablation study.

**Variant**	**GNN Layers**	**Transformer Blocks**	**Sequence Encoding**
Baseline	6	6	RiNALMo
V1	6	6	One-hot
V2	3	6	RiNALMo
V3	6	3	RiNALMo
V4	1	12	RiNALMo

3 Test set analysis

3.1 Distribution of Rfam families

To illustrate the diversity of our benchmark, we provide a complete breakdown of the 65 Rfam families represented in the test set (Table S4). Each entry

includes the Rfam ID, family name, and number of local descriptor structures. The number of representatives per family ranges from 6 to 500, reflecting natural imbalances commonly observed in structural RNA datasets. In addition, 4,359 structures in the test set are not assigned to any Rfam family but are included to ensure broader coverage of structural space beyond curated Rfam entries.

Table S4: Rfam families represented in the test set. The benchmark also includes 4,359 unannotated structures not assigned to any Rfam family.

No.	Rfam ID	Count	Family
1	RF00026	494	U6 spliceosomal RNA
2	RF00029	261	Group II catalytic intron
3	RF00167	204	Purine riboswitch aptamer
4	RF00010	153	Bacterial RNase P class A
5	RF00379	120	c-di-AMP riboswitch aptamer (ydaO/yuaA leader)
6	RF00174	101	Cobalamin riboswitch aptamer
7	RF00442	100	Guanidine-I riboswitch aptamer
8	RF00488	93	Yeast U1 spliceosomal RNA
9	RF00162	92	SAM riboswitch aptamer (S box leader)
10	RF00024	87	Vertebrate telomerase RNA
11	RF00059	82	TPP riboswitch aptamer (THI element)
12	RF00028	75	Group I catalytic intron
13	RF00011	67	Bacterial RNase P class B
14	RF01689	63	AdoCbl-II (Cobalamin/B12) riboswitch aptamer
15	RF02001	62	Group II catalytic intron D1-D4-3
16	RF00009	60	Nuclear RNase P
17	RF02678	60	Hatchet ribozyme
18	RF01750	56	ZMP/ZTP riboswitch aptamer (pfl RNA motif)
19	RF03013	49	nadA riboswitch
20	RF03167	49	c-di-GMP-I-GGC riboswitch aptamer
21	RF01856	46	Protozoan signal recognition particle RNA
22	RF00168	44	Lysine riboswitch aptamer
23	RF00234	43	glmS glucosamine-6-phosphate activated ribozyme aptamer
24	RF01831	42	THF (Tetrahydrofolate) riboswitch aptamer
25	RF00025	42	Ciliate telomerase RNA
26	RF00390	33	UPSK RNA
27	RF00619	33	U6atac minor spliceosomal RNA
28	RF01510	31	2dG-I riboswitch aptamer (M. florum)
29	RF02681	31	Twister_sister_ribozyme
30	RF01051	31	c-di-GMP-I riboswitch aptamer
31	RF00023	31	transfer-messenger RNA
32	RF01854	30	Bacterial large signal recognition particle RNA
33	RF00228	30	Hepatitis A virus internal ribosome entry site (IRES)

No.	Rfam ID	Count	Family
34	RF00525	30	Flavivirus DB element
35	RF04222	28	Flavivirus Potato leading responsible virus exoribonuclease-resistant RNA
36	RF00004	28	U2 spliceosomal RNA
37	RF00100	27	7SK RNA
38	RF01725	27	SAM-I/IV (S-adenosyl methionine) riboswitch aptamer
39	RF00050	25	FMN riboswitch aptamer (RFN element)
40	RF01734	24	Fluoride riboswitch aptamer (crcB RNA motif)
41	RF00380	24	M-box riboswitch aptamer (ykoK leader)
42	RF02683	24	NiCo (Nickel/Cobalt) riboswitch aptamer
43	RF00164	24	Coronavirus 3' stem-loop II-like motif (s2m)
44	RF02679	23	Pistol ribozyme
45	RF01786	23	Cyclic di-GMP-II riboswitch aptamer
46	RF00504	22	Glycine riboswitch aptamer
47	RF01344	20	CRISPR RNA direct repeat element
48	RF00458	19	Cripavirus internal ribosome entry site (IRES)
49	RF01415	18	Flavivirus 3'UTR stem loop IV
50	RF00030	15	RNase MRP
51	RF02519	14	ToxI antitoxin
52	RF03054	14	Xanthine riboswitch aptamer (NMT1 RNA)
53	RF01763	14	Guanidine-III riboswitch aptamer (yKKC-III)
54	RF01857	14	Archaeal signal recognition particle RNA
55	RF01739	14	Glutamine riboswitch aptamer (glnA leader)
56	RF04190	13	Hairpin ribozyme 1 from viruses-like metatranscriptomes
57	RF02796	12	Pab160 RNA
58	RF00066	12	U7 small nuclear RNA
59	RF02680	12	PreQ1-III (pre queuosine) riboswitch aptamer
60	RF01704	11	Glutamine-II riboswitch aptamer
61	RF01826	10	SAM-V (S-adenosyl methionine) riboswitch aptamer
62	RF02253	9	Iron response element II
63	RF00037	9	Iron response element I
64	RF00102	8	VA RNA
65	RF01357	6	CRISPR RNA direct repeat element
66	Other	4359	Structures not assigned to any Rfam family (e.g., 4L8H, 7MKY, 4QJD)

3.2 Distribution of test set descriptors across segment groups

Figure S1 shows the distribution of descriptor counts for each of the five segment groups in the test set. Each histogram (panels a–e) displays the frequency of descriptor occurrences within the corresponding group.

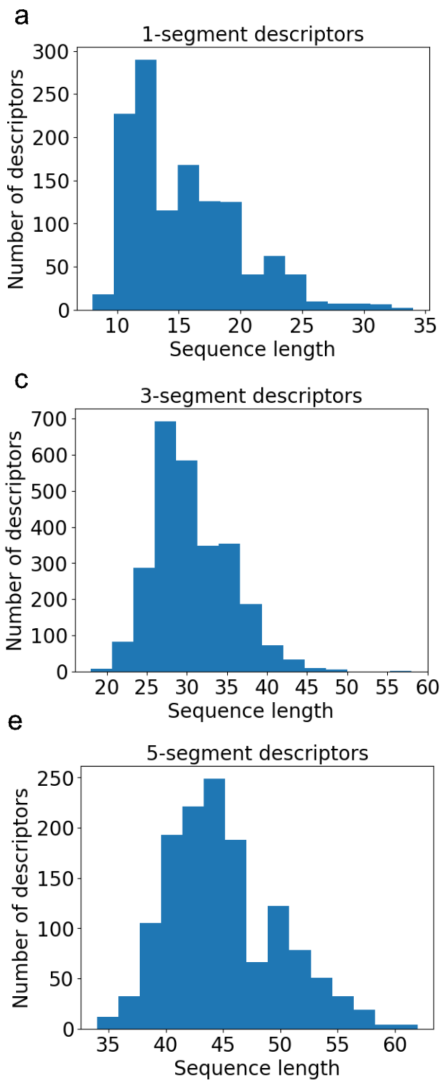

Figure S1: Distribution of descriptor counts across the five segment groups. Histograms (a–e) show the frequency of descriptor occurrences for groups containing 1 to 5 segments, respectively.

References

[1] Boniecki, M.J., Lach, G., Dawson, W.K., Tomala, K., Lukasz, P., Soltysinski, T., Rother, K.M., & Bujnicki, J.M. (2015) SimRNA: a coarse-grained method for RNA folding simulations and 3D structure prediction. Nucleic Acids Research, 44.

[2] Girshick, R. (2015) Fast r-cnn. In Proceedings of the IEEE international conference on computer vision (pp. 1440-1448).

[3] Rombach, R., Blattmann, A., Lorenz, D., Esser, P., & Ommer, B. (2022) High-resolution image synthesis with latent diffusion models. In Proceedings of the IEEE/CVF conference on computer vision and pattern recognition (pp. 10684-10695).

[4] Vaswani, A., Shazeer, N., Parmar, N., Uszkoreit, J., Jones, L., Gomez, A. N., ... & Polosukhin, I. (2017) Attention is all you need. Advances in Neural Information Processing Systems, 30.
